# A survey of egg-parasitoid species associated with *Dendrolimus houi* (Lepidoptera, Lasiocampidae) in Fujian, China

**DOI:** 10.3897/BDJ.14.e180701

**Published:** 2026-01-05

**Authors:** Ciding Lu, Qunda Chen, Xingying Li, Xu Han, Haoyu Lin, Huayan Chen, Guanghong Liang

**Affiliations:** 1 Forestry College, Fujian Agriculture and Forestry University, Fuzhou, China Forestry College, Fujian Agriculture and Forestry University Fuzhou China; 2 Fujian Forestry Vocational & Technical College, Nanping, China Fujian Forestry Vocational & Technical College Nanping China; 3 Forest Protection Research Institute, Fujian Academy of Forestry, Fuzhou, China Forest Protection Research Institute, Fujian Academy of Forestry Fuzhou China; 4 South China Botanical Garden, Chinese Academy of Sciences, Guangzhou, China South China Botanical Garden, Chinese Academy of Sciences Guangzhou China; 5 Fujian Agriculture and Forestry University, Fuzhou, China Fujian Agriculture and Forestry University Fuzhou China

**Keywords:** biological control, *Telenomus dendrolimi* (Matsumura), parasitoid, natural enemy

## Abstract

*Dendrolimus houi* Lajonquiere is a polyphagous defoliator with strong adaptability causing significant damage to hundreds of hectares of pine forest. The larvae primarily damage coniferous tree species. Given that the average female fecundity exceeds 300 eggs, population suppression prior to larval eclosion is critical to prevent massive infestations and minimise losses. The use of egg-parasitoids represents a biologically optimal control strategy, as they effectively attack non-migratory eggs and egg masses, while avoiding the drawbacks of chemical pesticides, such as resistance, residue and pest resurgence. Therefore, systematic field investigations are needed to identify and collect native dominant parasitoids of *D. houi* for subsequent mass rearing and release.

This study reports six parasitoid wasp species emerging from *D. houi* eggs: Eupelmidae: *Mesocomys menzeli* (Ferrière), *Mesocomys trabalae* (Yao et Yang), Anastatus (Anastatus) gastropachae Ashmead, Anastatus (Anastatus) meilingensis Sheng and Yu; Scelionidae: *Telenomus dendrolimi* (Matsumura); and Trichogrammatidae: *Trichogramma chilonis* Ishii. We compared key biological characteristics, including parasitism rate, longevity, offspring quantity and sex ratio, across all eight species. Based on this analysis, *T. dendrolimi*was identified as the dominant egg-parasitoid.

This study fills a critical gap in the systematic investigation of *D. houi* egg-parasitoids in Fujian, China. Our findings provide a scientific basis for the mass rearing and field release of these parasitoids, thereby supporting the biological control of *D. houi* in Chinese forests.

## Introduction

*Dendrolimus* spp. (Lepidoptera, Lasiocampidae), containing *D. punctatus* (Walker, 1855), *D. tabulaeformis* (Tsai et Liu, 1962), *D. spectabilis* (Butler, 1877), *D. houi* (Lajonquière, 1973), *D. superans* (Butler, 1879) and *D. kikuchii* (Matsumura, 1927) as well as other species, are major destructive defoliators of conifers in China (Qin et al. 2015). Both larvae and adults are capable of naturally spreading everywhere (Dang et al. 2018, Song et al. 2019) and annually destroy thousands of hectares of trees during their outbreaks (Zeng et al. 2010, Yang 2015). Notably, *D. houi* has caused infestation to over 100,000–200,000 ha of forests in China (Han et al. 2019), endangering Cryptomeria fortune var. sinensis Miq, *Cupressus funebris* Endl and *Pinus yunnanensis* Franch (Zhang and Li 2008, Miao et al. 2024).

Currently, chemical insecticides (e.g. deltamethrin, Sendebao: 0.18% abamectin + Bacillus thuringiensis at 10⁸ spores/g) are the primary means for controlling *D. houi* larvae. However, chemical control has several drawbacks, including short-lived efficacy and the risk of resistance development (Wang and Xia 2004), as well as public concern in ecologically sensitive areas, such as National Parks, Nature Reserves and Scenic Areas. Biological control is a more sustainable and effective alternative, particularly the use of parasitoid wasps that target eggs and early-instar larvae to suppress populations before outbreaks occur. In addition, *D. houi* harbours abundant parasitoid species as natural enemies with large wild populations and strong adaptability (Lin et al. 2018, Lin et al. 2023), indicating promising prospects for the development of biological control strategies. Over the past two decades, studies have systematically surveyed the pupal and larval parasitoid of *D. houi* and have developed mass-rearing methods for the pupal parasitoid wasps *Kriechbaumerella dendrolimi* Sheng & Zhong (Hymenoptera, Chalcididae), which has demonstrated effective pest suppression (Que et al. 2024). The eggs of *D. houi* are laid in relatively compact, immobile clusters during an early, prolonged egg stage of the life cycle, providing an optimal window for release of egg parasitoids. Egg parasitoids can prevent larval emergence by killing eggs, thereby averting the ecological and economic damage caused by larval defoliation. However, a lack of systematic surveys focused on egg parasitoids that attack the prolonged egg stage of *D. houi* has impeded the identification, screening and mass release of dominant egg‑parasitoid species; this knowledge gap is likely a major factor contributing to periodic outbreaks of *Dendrolimus* spp.

In this study, we collected and identified parasitoid species associated with *D. houi* eggs from *Cryptomeria japonica* forests. Collected eggs were reared under room conditions. During field surveys, we recorded parasitoid species, host‑forest distribution and parasitism rates. We then compared key developmental traits of recovered parasitoids, including offspring number (fecundity), sex ratio and development time, to identify dominant egg parasitoid species. These results provide a foundation for subsequent studies on their biology, mass rearing and field release.

## Material and methods

**Figure 1.**
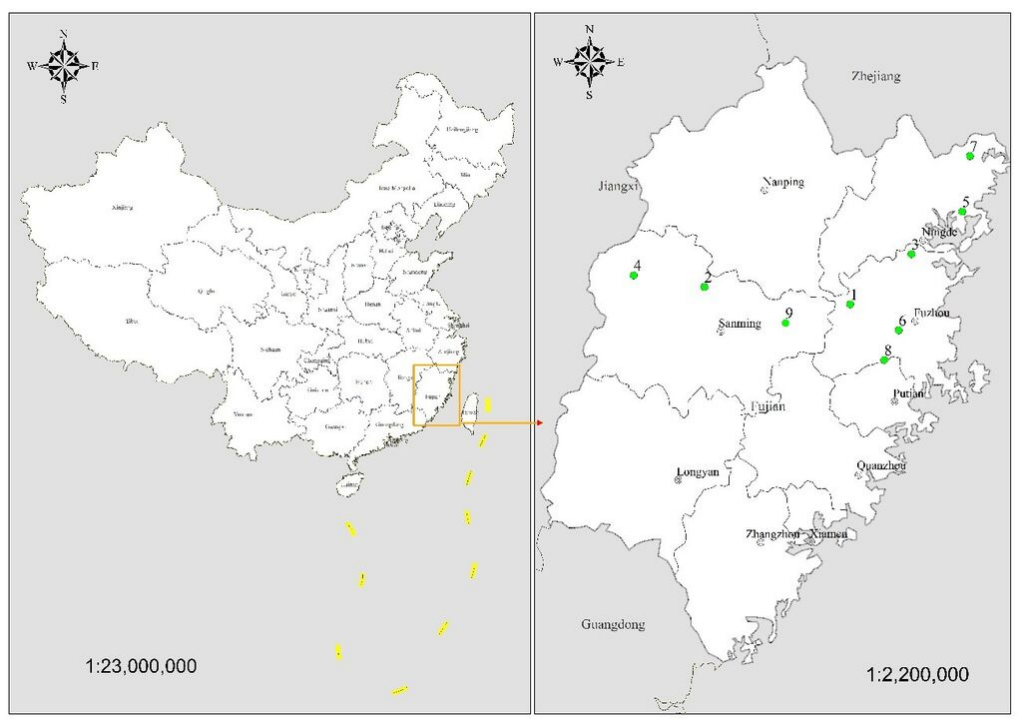
Green dots indicate sample sites of eggs in Fujian, China, 2019-2025. *Dendrolimus houi* was discovered at Sites 1 to 9. Geographical coordinates are as follows: Site 1 (MQB: Baiyunshan Forest Park, Minqing County); Site 2 (FJG: Guiyang Village, Jiangle County); Site 3 (LYS: Shangziyang, Luoyuan County); Site 4 (FJJ: Jinrao Moun, Jianning County); Site 5 (XPY: Yangmeiling Forest Park, Xiapu County); Site 6 (QSF: Qishan Forest Park, Fuzhou County); Site 7 (LSG: Liaoshan, Guanyang County); Site 8 (FYD: Duishan Village, Yongtai County) ; Site 9 (YXF: Youxi Forest Park, Youxi County).

Based on the distribution of *C. japonica* and its infestation by *D. houi*, nine representative sites in six cities of Fujian Province, China, were selected for investigation (Table 1). *Dendrolimus houi* eggs were collected throughout the entire oviposition period (October–March); individual eggs were labelled with unique identifiers and reared separately for subsequent analyses (Fig. 1).

Based on the previously reported outbreak areas of *D. houi* in Fujian Province, *D. houi* eggs were collected in October-December from the pure forests of *Cryptomeria fortune* which had experienced outbreaks (Fig. 2). The collected eggs were morphologically checked under a stereomicroscope, based on the description from previous publications to confirm they belonged to *D. houi*. Then, part of eggs from egg masses were reared under an artificial climate chamber (28℃, RH70%) until the 2^nd^ or 3^rd^ instar larvae, which can be easily identified as *D. houi* larvae. Additionally, eggs from female adults reared from collected eggs were also exposed to female *Telenomus dendrolimusi* to determine their parasitisation.

Fresh branches bearing *D. houi* eggs were collected and transported to the laboratory. The eggs were maintained at 25 ± 1°C, 50 ± 10% relative humidity (RH) and a 12:12 hour (L:D) photoperiod. After 25 days, emerged parasitoid adults were collected and housed in screened plastic boxes (16 × 9 × 6 cm) for biological observation. The parasitoids were provided with a 10% honey solution as a food source. Upon death, specimens were preserved in 75% ethanol. Morphological characteristics were examined and described using a stereomicroscope (SZ760B, Optec, Chongqing, China). Following preliminary identification, a subset of specimens was sent to senior taxonomists (Eupelmidae species were identified by Dr. Lingfei Peng of Fujian Agriculture and Forestry University, *Telenomus* species were identified by Dr. Huayan Chen of South China Botanical Garden, Chinese Academy of Sciences and *Trichogramma* species were identified by Dr. Naiquan Lin of Fujian Agriculture and Forestry University) for definitive species confirmation. Voucher specimens are deposited in the collection of Fujian Agriculture and Forestry University, Fuzhou, China.

Species of Eupelmidae were identified, based on morphological characters using the available keys present in Peng et al. (2020) and GIBSON (2021). *Telenomus* species were identified, based on morphological characters using the available keys present in Chen et al. (2024). *Trichogramma* species were identified, based on morphological characters using the available keys present in Huang (2003a).

Parasitoids were reared under controlled conditions (25 ± 2°C, 80 ± 10% RH and a 12:12 h L:D photoperiod). The number of emerging adults was recorded daily and individuals were sexed, based on morphological characteristics (presence of an ovipositor and body size). We evaluated the dominant parasitoid species by quantifying the following parameters: distribution, parasitism rate, offspring yield per host egg, sex ratio, longevity and fecundity (Borase et al. 2024, Ningthoujam et al. 2025).

The parasitism rate was calculated as (number of parasitized eggs/total number of eggs) × 100%. The sex ratio was expressed as the proportion of females (number of females/total adults). Longevity was defined as the period from adult emergence to death. Fecundity was measured as the mean number of offspring produced per female.

## Results

A total of six parasitoid species were identified, encompassing four genera, three families and one order: *Mesocomys trabalae*, *M. menzeli*, *Anastatus gastropachae*, *A. meilingensis*, *Telenomus dendrolimi* and *Trichogramma chilonis* (Table 2). Amongst these, two species — *A. meilingensis* and *T. chilonis* — are newly recorded as parasitoids of *D. houi* in Fujian Province, China.

A total of 5,268 *D. houi* eggs were collected from the field, with 508 (9.6%) being parasitised. Six parasitoid species emerged during the host egg stage across all nine sampling regions (Table 3). *Telenomus dendrolimi* was the most widely distributed, occurring in seven of the nine regions. In the YXF Region, it also exhibited the highest parasitism rate (25.93%). In contrast, *Trichogramma chilonis* produced a greater number of offspring per host egg than the other seven parasitoid species.

Based on key biological parameters — including field parasitism rate, developmental duration, distribution range, offspring yield per host and sex ratio (Table 4) — we conducted a preliminary screening of the candidate parasitoid species. *Trichogramma chilonis* and *Telenomus dendrolimi* exhibited the most favourable traits for biological control. Both species are polyparasitic, had the shortest developmental duration (14–16 days, compared to 45.32 days for *A. gastropachae*) and produced a high number of offspring, averaging over 25 adults per host egg. Furthermore, *T. dendrolimi* demonstrated a wide distribution in Fujian Province, indicating strong local adaptation and a highly female-biased sex ratio (> 89%), a critical factor for mass rearing and field establishment. Based on this comprehensive evaluation, *Telenomus dendrolimi* was selected as the dominant egg-parasitoid of *D. houi*.

## Discussion

Previous studies have documented 14 egg-parasitoid species of *D. houi*, spanning six families within a single order (Zhao et al. 1990, Lin et al. 2018,He and Tang 2024). In the present study, we identified eight parasitoid wasp species. This diversity provides a critical pool of natural enemy candidates for biological control, as it increases the likelihood of identifying species with both strong ecological adaptability and practical application potential.

The selection of a dominant natural enemy is typically based on criteria, such as host suitability, searching efficiency, environmental adaptability and parasitism rate (Du 1996). Additional assessment metrics include fecundity, phenological synchrony with the host and tolerance to environmental stressors (Baaren et al. 2024). Previous studies have selected dominant parasitoids for pests like *Sirex noctilio* and *Grapholita molesta* using indices of ecological dominance under natural conditions (Wang et al. 2021, Wang et al. 2024). In contrast, our objective was to identify a species suitable not only for survival in natural environments, but also for mass rearing. Consequently, we developed a comprehensive assessment system that scored species, based on both environmental adaptability (total parasitism rate, offspring yield, distribution) and rearing feasibility (sex ratio, longevity, developmental duration). Based on this system, *T. dendrolimi* was selected as the dominant egg-parasitoid of *D. houi*.

*Telenomus dendrolimi* was first recorded from a *Dendrolimus* sp. egg in Zhejiang Province, China, in 1934 and has since been reported across China and in other countries (Zhu 1955). While initially described as a specialist parasitoid of *Dendrolimus* eggs (He and Chen 2006), recent studies indicate a broader host range that includes species within the family Saturniidae (Chen et al. 2024). This plasticity in host use enhances its potential for sustained mass rearing and population stability in forest ecosystems.

*Trichogramma chilonis* is an important wasp in pest management and widely used worldwide (Wang et al. 2009, Visalakshi and Bhavan 2020). Laboratory observations showed that *T. chilonis* emerged approximately one day earlier than *T. dendrolimi* from multi-parasitised eggs. This developmental advantage may allow *T. chilonis* to pre-empt host resources, potentially explaining the failed eclosion of *T. dendrolimi*. However, in the high-altitude cultivation areas of Fujian host trees, *Trichogramma* species have a limited distribution (recorded in only three regions) and a low parasitism rate (0.8–4.3%). This suggests that habitat factors likely constrain *T. chilonis* population establishment and competitive impact in the field, a phenomenon supported by previous studies.

Similar multi-parasitism has been documented in other systems (Mohammadpour et al. 2014). For instance, the pupal parasitoids *Theronia depressa* and *Kriechbaumerella dendrolimi* can emerge from the same *D. houi* pupa (Lin et al. 2023) and multiple egg parasitoid species have been recorded from single hosts of other Lepidoptera (Li et al. 2018, Miao et al. 2024). These findings highlight the complex niche overlap and competitive interactions amongst parasitoids, which are critical to understanding their application in biological control.

## Conclusions

In summary, eight egg parasitoid species were collected from *Dendrolimus houi* across nine locations in Fujian Province. Through a comprehensive evaluation of both ecological and rearing parameters, *Telenomus dendrolimi* was identified as the most dominant and suitable egg parasitoid for future biological control efforts. These results directly provide information for large-scale biocontrol programmes by providing a science-based natural enemy candidate — its wide distribution, high parasitism rate and feasibility for mass rearing enable cost-effective field release in Fujian’s forest ecosystems, offering an environmentally friendly alternative to chemical pesticides and supporting sustainable forestry development. However, constraints include the need to verify its long-term colonisation efficiency under varying climatic and stand conditions, as well as its compatibility with other integrated pest management (IPM) strategies. Future research should address these gaps by investigating field release protocols, interspecific competitive dynamics in complex forest habitats and the impact of climate change on its biocontrol efficacy, to further optimise its application and promote ecological security in forest pest management.

## Figures and Tables

**Figure 2a.**
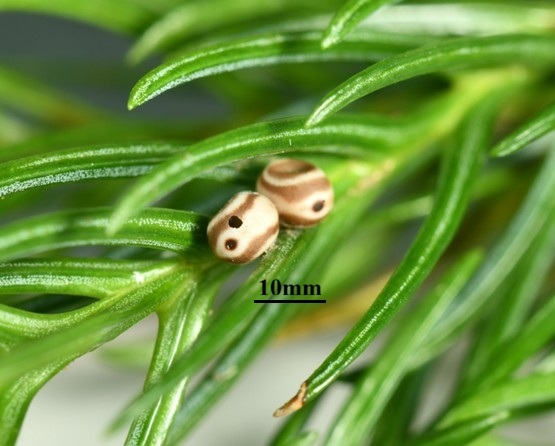
Eggs;

**Figure 2b.**
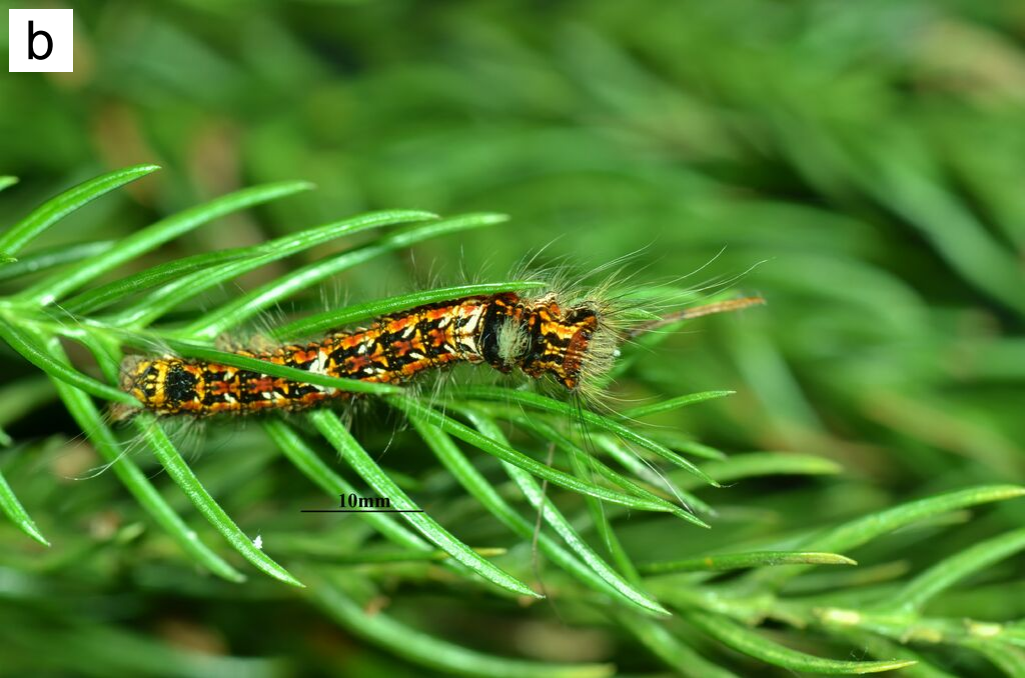
Larvae.

**Figure 3a.**
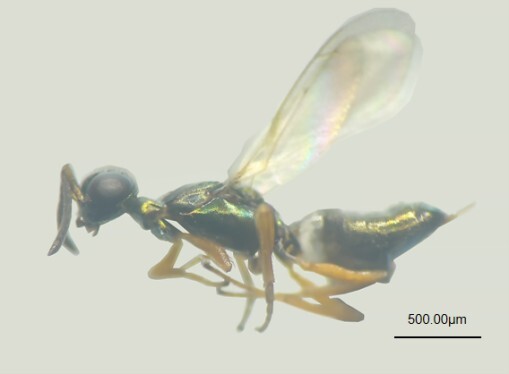
Female of *Mesocomys trabalae*;

**Figure 3b.**
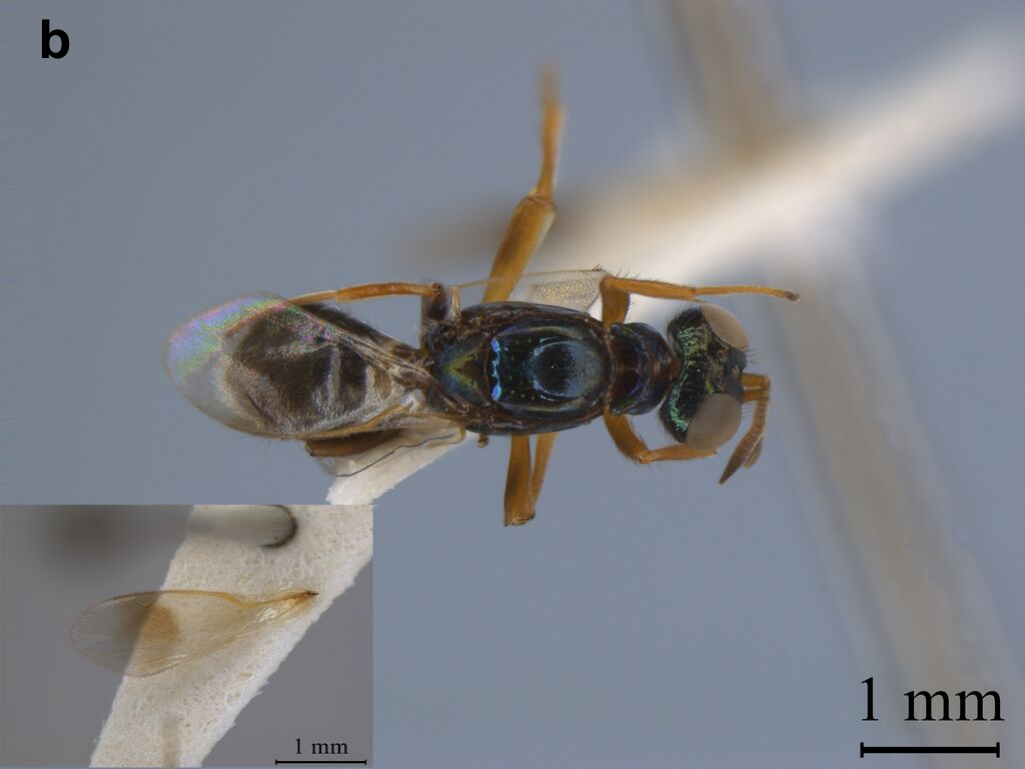
Female of *Mesocomys menzeli*;

**Figure 3c.**
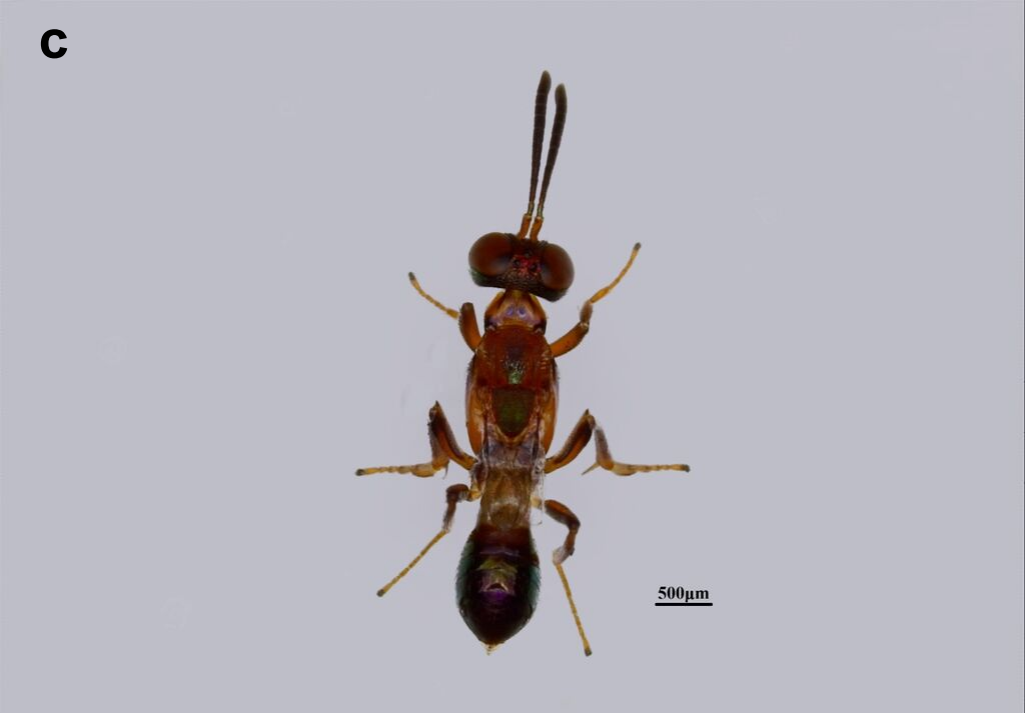
Female of *Anastatus gastropachae*;

**Figure 3d.**
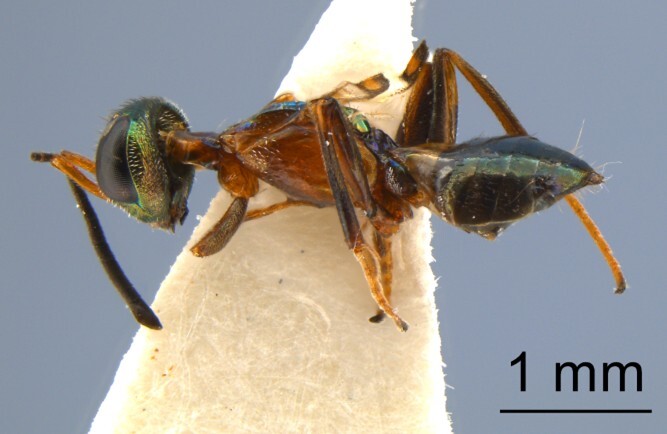
Female of *Anastatus meilingensis*.

**Figure 4a.**
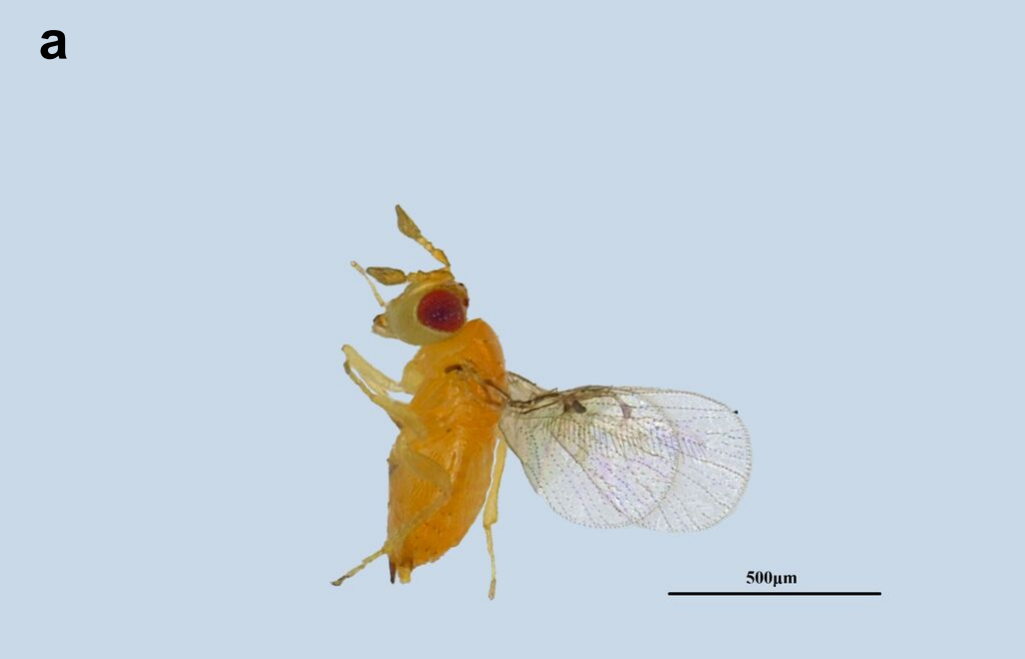
*Trichogramma chilonis*;

**Figure 4b.**
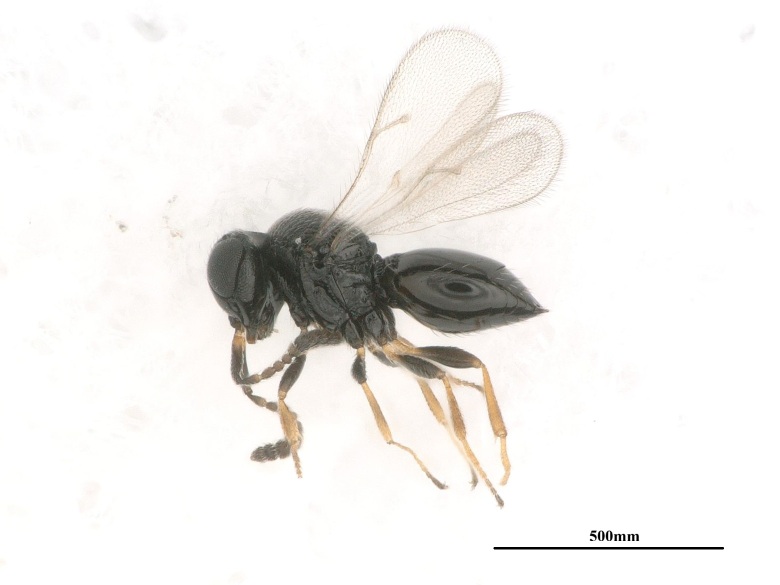
*Telenomus dendrolimi*;

**Table 1.** Summary of collections sites of *D. houi* forests in Fujian Province, China.

No.	Survey sites	Latitude/N, Longitude/E	Above Sea Level/m	City
1	Baiyunshan Forest Park (MQB)	26°15'38.30'', 118°46'29.65''	997	Minqing County, Fuzhou City
2	Jiangle (FJG)	26°35'33.23'', 117°31'17.18''	882	Guiyang Village, Jiangle County, Sanming City
3	Shangziyang (LYS)	26°34'7.55'', 119°23'31.49''	754	Luoyuan County, Fuzhou City
4	Jinrao Moun (FJJ)	26°46'21.14'', 116°54'49.59''	1019	Jianning County, Sanming City
5	Yangmeiling Forest Park (XPY)	26°49'43.27'', 119°54'26.86''	597	Xiapu County, Ningde City
6	Qishan Forest Park (QSF)	25°59'16.63'', 119°9'27.14''	787	Minhou Region, Fuzhou City
7	Liaoshan (LSG)	27°15'16.63'', 120°4'14.93''	903	Guanyang County, Ningde City
8	Yongtai (FYD)	25°46'29.49'', 118°59'8.37''	915	Duishan Village, Yongtai County, Fuzhou City
9	Youxi Forest Park (YXF)	26°12'7.76'', 118°10'55.15''	462	Youxi County, Sanming City

**Table 2.** Collected parasitoids species and their host species.

Order	Family	Species	Hosts
Hymenoptera	Eupelmidae	*Mesocomys trabalae* (Fig. 3a)	*D. houi*, *Trabala vishnou*, *Hippophae rhamnoides*, *Caligula japonica* (Yao et al. 2009, Yang et al. 2015, GIBSON 2021)
		*Mesocomys menzeli* (Fig. 3b)	*D. houi*, *Attacus atlas* (GIBSON 2021)
		*Anastatus gastropachae* (Fig. 3c)	*D. houi*, *D. punctatus*, *D. kikuchii*, *C. japonica*, *Antheraea pernyi* (Peng et al. 2020)
		*Anastatus meilingensis* (Fig. 3d)	*D. houi, *D. kikuchii*, *D. punctatus*, *Stauropus alternus*, *C. japonica* (Peng et al. 2020)
	Trichogrammatidae	*Trichogramma chilonis* (*Fig. 4a*)	*D. houi, *D. punctutus*, *D. kikuchii*, *Ostrinia furnacalis*, *Helicoverpa armigera*, *Plutella xylostella*, *Cnaphalocrocis medinalis* (Huang 2003a)
	Scelionidae	*Telenomus dendrolimi* (*Fig. 4b*)	*D. houi*, *D. punctutus*, *D. kikuchii* (Huang 2003b, Chen et al. 2024)

**Table 3.** Parasitism rate and distribution of parasitoids in eggs of *D. houi* in Fujian.

Species	Offspring	Parasitism rate (%) of egg-parasitoids in different locations								
		FJG	FJJ	LSG	XPY	QSF	YXF	MQB	LYS	FYD
*M. trabalae*	1~25		2.44	18.80				4.00		
*M. menzeli*	16~43	14.33								6.27
*A. gastropachae*	1~35		2.44				1.85	1.25	0.82	13.73
*A. meilingensis*	2~16			2.40			8.64	1.75	0.54	
*T. chilonis*	10~83			0.80	4.37	3.60				
*T. dendrolimi*	4~73			12.03	3.84	1.78	25.93	3.07	1.09	4.71

**Table 4.** Biological characteristics of parasitoid wasps.

Species	Total parasitism rates (%)	Offspring per host egg	Sex Ratio female: male	Female Longevity (d)	Development duration (d)	Number of Distributions
*M. trabalae*	8.41	2.67	0.33：1	34.67	32.46	3
*M. menzeli*	10.3	3.06	0.2：1	31.16	42.67	2
*A. gastropachae*	3.97	3.76	0.17：1	32.64	45.32	5
*A. meilingensis*	3.36	3.76	0.33：1	31.82	41.86	4
*T. chilonis*	2.96	26.36	4.26：1	27.67	14.76	3
*T. dendrolimi*	7.52	28.42	8.25：1	57.27	15.34	7
